# The Efficacy of Ozone Therapy on Pain and Soft Tissue Healing Associated With the Surgical Placement of Dental Implants: A Randomized Controlled Trial

**DOI:** 10.7759/cureus.85482

**Published:** 2025-06-06

**Authors:** Abhinav Shekhar, Chinmoy Sikdar, Shitij Srivastava, Anshuman Chaturvedi, Love K Bhatia, Debajyoti Sarkar

**Affiliations:** 1 Department of Prosthodontics, Sardar Patel Post Graduate Institute of Dental and Medical Sciences, Lucknow, IND

**Keywords:** dental implants, ozone, pain, randomized controlled trial, ­wound healing

## Abstract

Objectives

The primary objective of this randomized controlled trial was to evaluate the effect of ozone therapy on postoperative pain and the healing of soft tissues in patients undergoing dental implant placement.

Methods

This parallel-group, randomized clinical trial enrolled 84 participants requiring delayed implant placement. Participants were allocated by a computer-generated random sequence with allocation concealment by using the sequentially numbered, opaque, sealed envelopes (SNOSE) method. In group A, following local anesthesia and standard aseptic protocols, osteotomy was performed with irrigation using ozonated saline (25 μg/mL). Medical-grade ozone gas was introduced into the osteotomy site for 30 seconds, followed by peripheral ozone injection near the surgical site. Implants were placed with a torque of 35 Ncm, and the surgical site was closed with interrupted 3-0 silk sutures. In group B, conventional implant placement was performed using standard saline irrigation, with all other surgical steps, including implant brand, torque value, and suturing technique, kept identical to the experimental group. Postoperative pain was rated based on the visual analog scale at 24 and 48 hours; soft tissue healing was rated according to the Landry healing index at seven and 14 days. Outcome assessors were blinded to the group of the subjects, and data were analyzed using appropriate statistical methods, with a significance value considered at p < 0.05.

Results

The ozone therapy group showed a statistically significant reduction in pain scores at 24 hours and 48 hours compared to the control group (p < 0.05). In terms of soft tissue healing, the ozone group demonstrated improved healing rates, with earlier resolution of inflammation and faster epithelialization, particularly at 14 days postoperatively.

Conclusion

In the case of patients who have undergone dental implant surgery, ozone therapy seems to be a very good adjunct in relieving postoperative pain and promoting the healing of soft tissues. Consequently, these findings suggest that ozone therapy may be employed for enhancing patient recovery and improving clinical outcomes in implant dentistry.

## Introduction

Dental implants have become an accepted and effective means of treatment for replacing missing teeth [1,2]. Yet, postoperative complications like pain, swelling, and delayed soft tissue healing can reduce patient outcomes and postoperative recovery [3]. Generally, the management of pain following surgery incorporated with implants is achieved by using analgesics and anti-inflammatory drugs, which, being effective, may bring about some undesired side effects due to prolonged usage [4,5]. It is due to this reason that most of the researchers' interest has been directed toward other non-drug methods that would enhance the possible improvement of healing in soft tissues and reduction in post-surgery discomfort [6].

Using ozone (O₃) therapy is emerging as a potent adjunctive dental treatment procedure for its antimicrobial, anti-inflammatory, and regenerative properties [7]. Ozone, a highly reactive type of oxygen, has proven to speed up wound healing, oxygenate tissues, and boost immunity, thus becoming a promising agent for post-surgical recovery enhancement [8,9]. With this in mind, in implantology, ozone therapy could be employed in the postoperative period to alleviate pain, prevent infections, and allow soft tissue healing to take place more quickly [10].

While there are many applications for ozone in medicine, especially in dentistry, its importance in dental implant surgery remains unexplored [11]. While some studies seem to indicate high success rates with ozone therapy for general wound healing, hardly any evidence is available on examining pain relief and soft tissue healing during implant placement [12,13]. Judging by the above reasons, there is a need for multiple rigorous clinical trials regarding the evaluation of ozone therapy as an adjunct to standard postoperative care in dental implants [14].

This research could aptly solicit or fill that gap by conducting a randomized controlled trial on effective ozone therapy as compared to control treatment about pain relief and healing of soft tissues after the surgical placement of dental implants [15]. Thus, it will contribute to the evidence, potentially informing future clinical practices in implant dentistry, comparing the patients receiving ozone therapy with those undergoing conventional treatment [16].

Increased demand for dental implants as a definitive solution to tooth loss has made advancement in postoperative care essential to maximizing success [2]. The most significant problem that confronts professionals and their patients is pain management after surgical placement of dental implants [17]. Postoperative pain includes inflammation and tissue healing with increased patient discomfort and prolonged recovery, as well as the increasing chances of implant failure in some cases [18]. These complications arise, necessitating the entry of new dimensions in treatment for reducing pain and hastening the process.

Traditionally, pain in post-surgical implant cases has been managed through pharmacologic agents like analgesics or nonsteroidal anti-inflammatory drugs (NSAIDs) [19]. Though these medications work, most of their side effects include gastrointestinal problems, allergic reactions, and sometimes dependence after long-term use [9]. Moreover, anti-inflammatory drugs may sometimes interfere with the natural healing process by inhibiting the necessary inflammatory response that facilitates the repair of tissues [10]. Therefore, alternative approaches to treat pain and healing of soft tissue should be developed that are completely non-invasive and have low risk owing to pharmaceuticals.

The therapy that ozone provides is extremely promising due to the well-documented antimicrobial, anti-inflammatory, and regenerative properties [12]. Ozone favors the healing of wounds by stimulating oxygen metabolism through the improvement of blood circulation and immune response [13]. Keeping bacteria and other pathogens in check, ozone's strong oxidizing power reduces the chances of postoperative infections that harm the healing process. Apart from this, the property of reducing oxidative stress and modulating inflammation makes ozone an effective agent in the management of postoperative pain and regeneration of tissue after surgeries like those in dental implantology [15].

This study aims to evaluate the efficacy of ozone therapy as an adjunct to conventional postoperative care in reducing pain and enhancing soft tissue healing following dental implant surgery. It is hypothesized that the adjunctive use of ozone therapy will significantly reduce postoperative pain and promote faster and more effective soft tissue healing compared to conventional postoperative care alone.

## Materials and methods

Ethical approval

The clinical trial was conducted according to the ethical standards laid down in the Declaration of Helsinki. The study protocol received ethical clearance from the Institutional Ethical Committee (PROSTHO/03/222333/IEC) and was prospectively registered with the Clinical Trials Registry-India (CTRI/2023/09/057321). Written informed consent was obtained from all participants before inclusion, and participants were informed of their right to withdraw at any stage without any consequence to their treatment.

Study design

This was a randomized, in vivo, experimental clinical trial following a parallel group design. Participants were randomly allocated into two groups: group A (experimental group) - ozone-treated implants; group B (control group) - standard implant procedure.

**Figure 1:**
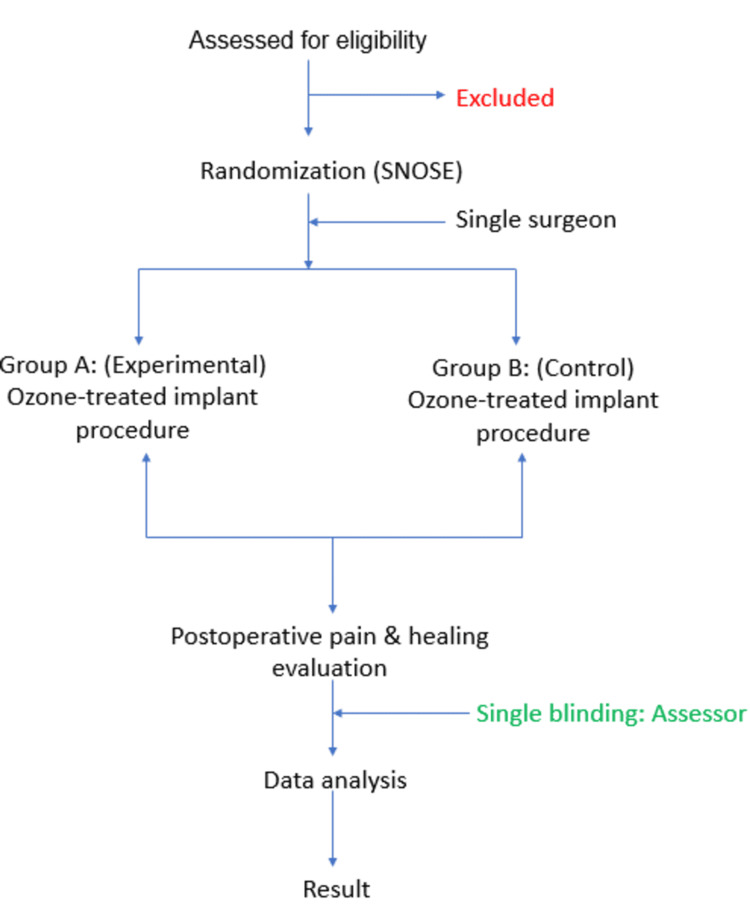
Study design flowchart. SNOSE: sequentially numbered, opaque, sealed envelopes.

Randomization was performed using a computer-generated sequence, and allocation concealment was ensured using sequentially numbered, opaque, sealed envelopes (SNOSE). Blinding was implemented for outcome assessors and data analysts to minimize bias. Due to the nature of the intervention, the operating surgeon was not blinded. However, postoperative pain and healing evaluations were conducted by a single calibrated examiner blinded to group assignment (Figure 1).

Sample selection

Participants included those with D2-D3 bone quality in both arches, indicated for delayed implant placement, in good physical and mental health, and possessing a well-formed or moderately formed residual alveolar ridge. Individuals were non-smokers, had not undergone periodontal treatment within the previous year, had not taken systemic antibiotics for the past six months, were neither pregnant nor lactating, and were suitable candidates for both dental implant surgery and ozone therapy.

Participants excluded from the study were individuals with systemic medical conditions, candidates for immediate implant placement, or those with temporomandibular disorders, severe ridge resorption, orofacial tumors, gross facial deformities, mucositis, history of radiation therapy, or parafunctional habits. Participants with glucose-6-phosphate dehydrogenase deficiency (favism), which contraindicates ozone therapy, and those with compromised immune function were also excluded.

Sample size calculation

The sample size for the present study was estimated based on data from a previous study by Shekhar et al. (2021) [20]. This study evaluated ozone therapy regarding pain intensity by using the visual analog scale (VAS) on day seven and had come up with a mean difference of 1.14 between groups. The sample size was worked out by using the formula proposed by Charan and Biswas (2013) [21]. Hence, based on the approach given, the sample size calculated was 38 in each group. Compensating for data loss of about 10% and to maintain even distribution, the final proposed minimum sample was 42 in each group, i.e., a total of 84 for the study.

Surgical procedure

This randomized, parallel-group clinical trial was held in the department of prosthodontics from October 2023 to December 2024. All participants underwent the placement of MIS Seven implants (MIS Implants Technologies Ltd., Misgav, Israel), which are tapered, self-tapping, screw-type titanium implants with a sandblasted and acid-etched (SLA) surface optimized for primary stability. The implants were selected according to individual bone volume and quality assessed preoperatively.

Group A: Ozone-Treated Implants (Experimental Group)

After administration of local anesthesia and following aseptic procedures, a mid-crestal incision with a sulcular extension was made with a No. 15 BP blade. Full-thickness mucoperiosteal flaps were raised. Initial osteotomy was done using a No. 5 round bur, followed by sequential drilling with copious irrigation of ozonated saline (25 μg/mL) instead of normal saline. Medical-grade ozone gas was then infused directly into the osteotomy site through a bent ozone needle for 30 seconds, with high-volume suction employed to avoid its inhalation. Peripheral injection of ozone was given adjacent to the osteotomy site to improve soft tissue response. Implants (MIS Seven) were placed at a torque of 35 Ncm, and the flap was repositioned and sutured closed using interrupted 3-0 silk sutures .

Group B: Standard Implants (Control Group)

The procedure followed the conventional implant placement protocol, including a mid-crestal incision, flap elevation, and osteotomy preparation using standard drills and sterile saline irrigation. MIS Seven implants were placed under the same torque value (35 Ncm) and sutured in the same manner.

Pain assessment

Postoperative pain was evaluated using the VAS, where participants indicated their pain level on a 100 mm line. Interpretation of VAS scores was categorized as follows: 0-4 mm: no pain; 5-44 mm: mild pain; 45-74 mm: moderate pain; 75-100 mm: severe pain.

Pain assessments were recorded at specific time intervals post surgery to monitor the progression and intensity of discomfort experienced by participants.

Soft tissue healing assessment

Soft tissue healing around the dental implants was evaluated clinically based on tissue color, bleeding on palpation, granulation tissue, epithelialization, and presence of suppuration. The healing index developed by Landry, Turnbull, and Howley was used to grade healing outcomes, with scores ranging from 1 to 5 as follows: score 1 (very poor): ≥50% of gingiva red, bleeding on palpation, granulation tissue present, incision margin not epithelialized, suppuration present; score 2 (poor): ≥50% of gingiva red, bleeding on palpation, granulation tissue present, incision margin not epithelialized, connective tissue exposed; score 3 (good): 25%-50% of gingiva red, no bleeding, no granulation tissue, no connective tissue exposed; score 4 (very good): <25% of gingiva red, no bleeding, no granulation tissue, no connective tissue exposed; score 5 (excellent): all tissues pink, no bleeding, no granulation tissue, no connective tissue exposed.

Assessments were performed at predetermined follow-up visits to monitor healing progression between both groups.

Data collection procedure and statistical tools

Data on the efficacy of ozone therapy in improving postoperative outcomes following dental implant placement were collected using standardized, pre-approved forms to ensure consistency and accuracy. Upon recruitment, participants were randomly assigned to either the ozone therapy group or the control group. The control group received conventional saline irrigation, while the experimental group was treated with ozone in three forms: ozonated saline, ozone gas, and peripheral ozone injections at the implant site.

Postoperative assessments included pain evaluation using the VAS at 24 and 48 hours and soft tissue healing assessment using the Landry healing index at seven and 14 days.

All data were entered into each participant’s clinical chart and then transcribed into secure, password-protected digital files. Data entry was performed independently by two trained personnel and cross-verified for accuracy. Any adverse events were recorded and managed according to standard clinical protocols. All outcome assessments were performed by a calibrated, blinded examiner, achieving an intra-examiner kappa value of 0.88 prior to study commencement.

All statistical analyses were done using SPSS version 21.0 (IBM Corp., Armonk, NY). Continuous variables were presented as mean ± standard deviation (SD), while categorical variables were presented as numbers (percentage). Intergroup comparisons were made using parametric or non-parametric analysis according to the statistical distribution of the data. Values of p < 0.05 were considered significant.

The study's statistical analysis consisted of descriptive and inferential methods. Descriptive statistics, including mean, standard deviation, frequency, and percentage, were used to give summary statistics for variables related to demographics, such as age, gender, distribution of implant sites, and some clinical variables, such as pain scores and indices of tissue healing. The inferential statistical techniques included the chi-square tests for comparing categorical variables like age group distribution, gender distribution, and implant placement sites of the two groups, which revealed no statistically significant difference, and hence can be said that the two groups were comparable at the baseline. The continuous variables, such as pain scores and indices of tissue healing, were analyzed using an independent samples Student’s t-test.

## Results

Out of 84 patients, 42 (50.0%) patients were randomly allocated to group A in whom implant was placed after topical application of ozone gas, ozonated saline and ozone injection of 25 μg/ml concentration at osteotomy sites, and administration of ozone injection locally near the osteotomy site, rest 42 (50.0%) patients were allocated to group B wherein implants were placed according to standard protocol with profuse irrigation with normal saline (Table 1).

**Table 1:** Group-wise distribution of the study population.

S. No.	Group	Intervention	No.	%
1	Group A	Ozonated water	42	50.0
2	Group B	Normal saline	42	50.0
	Total		84	100.0

All patients were aged ≥18 years. Though the proportion of younger patients was higher in group B as compared to group A, i.e., 18-25 years (14.3% vs. 7.1%) and 26-35 years (31.0% vs. 21.4%), yet this difference was not found to be statistically significant (p = 0.504; Table 2).

**Table 2:** Intergroup comparison of age of the study population The table shows the number (No.) and percentage (%) of participants across different age groups in both groups. Statistical analysis using the chi-square test indicates no significant difference in age distribution between groups (χ² = 3.329, p = 0.504).

S. No.	Age group	Group A		Group B	
		No.	%	No.	%
1	18-25 years	3	7.1	6	14.3
2	26-35 years	9	21.4	13	31.0
3	36-45 years	13	31.0	12	28.6
4	46-55 years	6	14.3	5	11.9
5	≥56 years	11	26.2	6	14.3
χ^2 ^= 3.329; p = 0.504					

Out of 84 patients enrolled in the study, 42 were males and 42 were females. In group A, the proportion of females was 52.7%, while in group B, the same was 47.6% only; the rest of the patients in the above two groups were males. The difference in gender of patients in the above two study groups was not found to be statistically significant (p = 0.513; Table 3).

**Table 3:** Intergroup comparison of gender of the study population. The table presents the number (No.) and percentage (%) of male and female participants in each group. Chi-square analysis showed no significant difference in gender distribution between groups (χ² = 0.429, p = 0.513).

S. No.	Gender	Group A		Group B	
		No.	%	No.	%
1	Female	22	52.4	20	47.6
2	Male	20	47.6	22	52.4
χ^2 ^= 0.429; p = 0.513					

Most common site of implant placement in group A was second molar (M-2, 26.2%), followed by first molar (M-1, 23.8%), central incisor (CI, 21.4%), and second premolar (PM-2, 19.0%), while in group B, the most common site of implant placement was PM-2 (26.2%), followed by M-2 (23.8%) and CI & M-1 (21.4% each). In both groups, the least common sites of implant placement were LI (2.4% each) and PM-1 (7.1% in group A and 4.8% in group B). The difference in site of implant placement in the above two study groups was not found to be statistically significant (p = 0.979; Table 4).

**Table 4:** Intergroup comparison of the site of implant. The table lists the number (No.) and percentage (%) of implants placed at different tooth sites for both groups: central incisor (CI), lateral incisor (LI), first molar (M-1), second molar (M-2), first premolar (PM-1), and second premolar (PM-2). Chi-square test showed no statistically significant difference in implant site distribution between groups (χ² = 0.774, p = 0.979).

S. No.	Site	Group A		Group B	
		No.	%	No.	%
1	CI	9	21.4	9	21.4
2	LI	1	2.4	1	2.4
3	M-1	10	23.8	9	21.4
4	M-2	11	26.2	10	23.8
5	PM-1	3	7.1	2	4.8
6	PM-2	8	19.0	11	26.2
χ^2 ^= 0.774; p = 0.979					

Pain score of group B was found to be significantly higher than that of group A at 24 hours post implant placement (77.83 ± 3.96 vs. 57.81 ± 4.09), 48 hours post implant placement (60.83 ± 4.13 vs. 37.86 ± 4.16), and at day seven (38.81 ± 2.46 vs. 4.02 ± 4.94) (Table 5).

**Table 5:** Intergroup comparison of pain as measured by VAS at different observations. Pain levels were measured using the visual analog scale (VAS) at 24 hours, 48 hours, and day seven post surgery. Values are expressed as mean ± standard deviation (SD). Student’s t-test was used to compare groups, showing statistically significant lower pain scores in group A at all time points (p < 0.001).

S. No.	Time	Group A		Group B		Student’s t-test	
		Mean	SD	Mean	SD	‘t’	‘p’
1	24 hours	57.81	4.09	77.83	3.96	-22.765	<0.001
2	48 hours	37.86	4.16	60.83	4.13	-25.401	<0.001
3	Day 7	4.02	4.94	38.81	2.46	-40.837	<0.001

The tissue healing index of group A patients was significantly higher than that of group B on day seven (4.48 ± 0.55 vs. 3.14 ± 0.42) and day 14 (4.93 ± 0.26 vs. 4.07 ± 0.26) (Table 6).

**Table 6:** Intergroup comparison of tissue healing at different intervals. Healing scores are expressed as mean ± standard deviation (SD). Statistical comparison using Student’s t-test revealed significantly better soft tissue healing in group A at both time points (p < 0.001).

S. No.		Group A		Group B		Student’s t-test	
		Mean	SD	Mean	SD	‘t’	‘p’
1	Day 7	4.48	0.55	3.14	0.42	12.492	<0.001
2	Day 14	4.93	0.26	4.07	0.26	15.069	<0.001

## Discussion

The present investigation was conducted to evaluate the utility of ozone therapy in enhancing soft tissue healing after dental implant surgery and in managing postoperative pain. The findings significantly prove the efficacy of ozone treatment as the healing had a better outcome in the ozone-treated group than in the control one, both at seven and 14 days post operation, thus supporting the hypothesis that ozone can complement implant dentistry practice effectively.

Ozone therapy has provided a promising therapeutic option, having anti-microbial, anti-inflammatory, and biostimulatory effects in medicine and dentistry. In the present study, ozone application around the implant site resulted in increased healing index scores, most likely due to oxygenation, decreased microbial load, and stimulation of local immune and regenerative responses. This finding supports previous work done, which demonstrated that ozonated water benefits periodontal therapy by improving fibroblast activity and angiogenesis [22].

The biological mechanisms that underlie the efficiency of ozone include the production of reactive oxygen species (ROS) and lipid oxidation products (LOPs), which act like messengers to stimulate and provide signals for wound healing, inflammatory process modulation, and growth factor release. The mossy healing process may be due to a reduction of the same inflammation and faster healing that can be imparted by enhanced neovascularization and activation of immune cells.

All the available data appear to recommend the idea that ozone therapy would be expected to improve postoperative outcomes in implant dentistry. The results of this dual action component, antimicrobial and tissue-regenerative, recommend the use of ozone therapy as an adjunct to conventional protocols. More clinical trials need to be conducted with larger sample sizes and longer follow-up times to validate and increase the weight of this finding.

These findings are supported by earlier studies on ozone therapy, establishing its role in soft tissue healing and pain relief after surgery. Ozone irrigation results in low pain perception and fast healing when compared to saline, owing to ozone's anti-inflammatory and antimicrobial properties [15]. This observation was consistent with the current study, which reported reduced VAS scores and improved healing indices.

Ozone's effect on peri-implant tissues and its regenerative potential provide a favorable environment for wound healing by controlling microbial load and enhancing oxygenation [20]. Ozone's diverse biological actions, including improving oxygen delivery and modulating immunity, support the observations in our ozone-treated group for better healing.

The effect of ozone on the stimulation of fibroblasts during oral surgery, and less postoperative pain, agrees with our results of faster recovery and better pain control of patients [16].

Thus, all mentioned studies highlight a collective reinforcement of our conclusion. Ozone therapy, in its antimicrobial, anti-inflammatory, and regenerative properties, has been shown to have a significant impact on postoperative outcomes of dental implant surgery.

Limitations of the study

From a promising standpoint for adjunctive ozone therapy, one must consider its limitations. First, the short follow-up of 14 days limits our insight into the long-term soft tissue healing around implants and implant success. Second, although statistically justified, larger multicenter studies will yield somewhat generalizable and externally valid results. Third, surgeons were not blinded to the intervention; thus, performance bias may have been introduced, whereas outcome assessors were blinded to minimize assessment bias. Several factors were not assessed in the study, such as peri-implant bone level changes, and other patient-reported outcomes besides pain perception. Finally, technique sensitivity and standardization of ozone delivery protocols should be considered as additional variables affecting the reproducibility of the studies in different clinical setups.

## Conclusions

This study shows how ozone therapy might promote successful dental implantation. Ozone treatment significantly reduced postoperative pain measured by VAS scores and improved soft tissue healing, noted in higher healing index scores against saline. This is due to ozone promoting postoperative healing through its antimicrobial, anti-inflammatory, and tissue-regenerative actions, all widely reported in the literature. Inclusion of ozone in implant protocols holds promise to enable faster healing and fewer complications, hence better patient outcomes. These findings should be tested by further clinical research with a larger patient population to see if such implications sustain over the long term in implant dentistry.
